# The Prognostic Value of Early Detection of Minimal Residual Disease as Defined by Flow Cytometry and Gene Mutation Clearance for Myelodysplastic Syndrome Patients After Myeloablative Allogeneic Hematopoietic Stem-Cell Transplantation

**DOI:** 10.3389/fonc.2021.700234

**Published:** 2021-08-05

**Authors:** Chang Hou, Lili Zhou, Menglu Yang, Shuhui Jiang, Hongjie Shen, Mingqing Zhu, Jia Chen, Miao Miao, Yang Xu, Depei Wu

**Affiliations:** ^1^Jiangsu Institute of Hematology, National Clinical Research Center for Hematologic Diseases, The First Affiliated Hospital of Soochow University, Suzhou, China; ^2^Institute of Blood and Marrow Transplantation, Collaborative Innovation Center of Hematology, Soochow University, Suzhou, China

**Keywords:** myelodysplastic syndromes (MDS), allogeneic hematopoietic stem cell transplantation (AHSCT), minimal residual disease (MRD), multiparameter flow cytometry (MFC), gene mutation clearance

## Abstract

High relapse incidence remains a major problem for myelodysplastic syndrome (MDS) patients who have received an allogeneic hematopoietic stem-cell transplantation (allo-HSCT). We retrospectively analyzed the correlations between clinical outcomes and minimal residual disease (MRD) by using mutations (MUT) and flow cytometry (FCM) analysis of 115 MDS patients with allo-HSCT. We divided 115 MDS patients into four groups based on molecular genetics and FCM MRD results at day 30 post-HSCT. There were significant differences in the 2-year progression-free survival (PFS) between the FCM^high^ MUT^pos^ and FCM^low^ MUT^neg^ groups (20% vs 79%, P < 0.001). In addition, by univariate analysis, we found that an IPSS-R score ≥4 pre-HSCT (HR, 5.061; P=0.007), DNMT3A mutations (HR, 2.291; P=0.052), TP53 mutations (HR, 3.946; P=0.011), and poor and very poor revised International Prognostic Scoring System (IPSS-R) cytogenetic risk (HR, 4.906; P < 0.001) were poor risk factors for PFS. In multivariate analysis, we found that an IPSS-R score ≥ 4 pre-HSCT (HR, 4.488; P=0.015), DNMT3A mutations (HR, 2.385; P=0.049), positive FCM MRD combined with persistence gene mutations at day 30 (HR, 5.198; P=0.013) were independent risk factors for disease progression. In conclusion, our data indicated that monitoring MRD by FCM combined with gene mutation clearance at day 30 could help in the prediction of disease progression for MDS patients after transplantation.

## Introduction

Myelodysplastic syndromes (MDSs) are myeloid neoplasms with highly variable clinical survival outcomes that depend on several prognostic scoring systems based on clinical/hematological parameters (1). Allogeneic hematopoietic stem-cell transplantation (allo-HSCT) is the only curative therapy for MDS patients diagnosed as being intermediate and high risk according to the revised International Prognostic Scoring System (IPSS-R) and WHO classification-based prognostic scoring system (WPSS) (2, 3). As a usual conditioning regimen, standard myeloablative conditioning (MAC) could effectively eliminate blast cells. However, it is reported that the incidence of relapse in MDS patients who received MAC allo-HSCT ranged from 14% to 50% (4–6), thus it was essential to monitor minimal residual disease (MRD) regularly. The current detection method of MRD is based on polymerase chain reaction (PCR), multiparametric flow cytometry (MFC), and gene mutation burden, which can assess different time points and provide useful information in patients with myeloid malignancies undergoing allo-HSCT. Several researchers used MFC to detect MRD in MDS after therapy (1, 2). However, the MFC still has its drawbacks, including a lack of standardized inter-lab leukemia-associated aberrant immunophenotypes (LAIPs) (3). What’s more, it should be noted that the percentage of blast cells in MDS patients before HSCT was usually more than 5%; thus, it also remains unclear when we should carry out MRD detection. Some studies suggested the presence of MRD as determined by FCM at 30 days post-HSCT was likely regarded as a marker to identify subgroups of patients (3, 4). In general, the timing, sensitivity, and specificity of MRD monitoring for MDS patients with allo-HSCT still need to be further verified.

Currently, somatic mutations are common in more than 75% of MDS patients, who presented shorter OS than those without prognostic mutations (5–7). A prior study identified mutations in TP53, TET2, DNMT3A, JAK2, and RAS pathway were associated with shorter OS after transplantation (8, 9), and the presence of U2AF1, high clone burden of EZH2, and TP53 mutations were associated with poor relapse-free survival (RFS) in the context of HSCT (10). Of note, the impact of TP53 mutation was independent of the IPSS-R, ASXL1, and RUNX1 mutations (11). Furthermore, prior studies showed that MDS and MDS/MPN patients with detectable molecular mutations at post-HSCT might result in a higher incidence of relapse than those without mutations (12). Besides, the risk of disease progression was higher among MDS patients who had mutations with a higher maximum variant allele frequency (VAF) after HSCT than those who did not (13). Therefore, the value of molecular minimal residual disease needs to be explored. It was reported that the clearance of somatic gene mutations after HSCT might reflect the number of MDS clones but could also reflect the sensitivity of MDS clones to the intensity of the conditioning regimen (13). However, the value of mutation clearance in MRD monitoring in MDS patients has not been clarified. Interestingly, the combination of the expression of the Wilms tumor 1 (WT1) gene and the presence of FCM-MRD was already used as a risk factor for the prediction of disease progression in MDS patients (14, 15). The predictive value of mutation clearance and FCM in MDS patients with allo-HSCT needs to be further explored.

In the study presented here, we retrospectively analyzed the correlations between clinical outcomes and the molecular genetics and FCM MRD results of 115 MDS patients with MAC allo-HSCT. In particular, our study showed that 30 days post-HSCT could be a suitable time point for MRD monitoring.

## Method

### Patients

This is a retrospective study based on the transplantation database at our center. As our center is a member of the European Society for Blood and Marrow Transplantation (EBMT), our database is designed in accordance with the requirements of the EBMT registry. A total of 115 MDS patients at the First Affiliated Hospital of Soochow University between June 1, 2016, and November 31, 2019, were brought in if they met the following criteria: (1) patients who were 10 to 65 years old, (2) patients having at least one mutation detected by next-generation sequencing (NGS) at their initial disease diagnosis or pre-HSCT, and (3) patients receiving a MAC protocol before HSCT. Characteristics of the 115 patients are summarized in **Table 1**. The median age was 43 years (range 13–64); 73 (63.5%) patients were male, and 42 (36.5%) patients were female. Disease was classified as SLD in 3 (2.6%) patients, MLD in 19 (16.5%) patients, RS-MLD in 2 (1.7%) patients, EB-1 in 34 (29.6%) patients, and EB-2 in 57 (49.6%) patients. The median (range) number of blast cells in bone marrow (BM) at diagnosis was 8.5% (2%~19%).

**Table 1:** Clinical characteristics of patients.

Characteristics	
**No. of patients (%)**	115
**Age at diagnosis, median (range)**	43 (13-64)
**Sex at diagnosis, n (%)**	
**male**	73 (63.5)
**female**	42 (36.5)
**diagnosis classification (WHO 2016), n (%)**	
**SLD**	3 (2.6)
**MLD**	19 (16.5)
**RS-MLD**	2 (1.7)
**EB-1**	34 (29.6)
**EB-2**	57 (49.6)
**IPSS-R cytogenetic risk at diagnosis, n (%)**	
**Very good/Good**	69 (60.0)
**Intermediated**	35 (30.4)
**Poor/Very poor**	11 (9.6)
IPSS* risk at diagnosis, n (%)	
**Low**	1 (0.9)
**Intermediate -1**	48 (41.7)
**Intermediate-2**	47 (40.9)
**High**	19 (16.5)
IPSS-R* risk at diagnosis, n (%)	
**Very low**	0
**Low**	7 (6.1)
**Intermediate**	32 (27.8)
**High**	47 (40.9)
**Very high**	29 (25.2)
**Therapy before HSCT**	
**Induction Chemotherapy**	43 (37.4)
**HMA**	50 (43.5)
**No chemotherapy**	22 (19.1)
**CR at pre-HSCT**	59 (51.3)
**Time from diagnosis to HSCT (months)**	
**<3**	45 (39.1)
**3~12**	59 (51.3)
**≥12**	11 (9.6)
**HLA matched, n (%)**	48 (41.7)
**Source of stem cell, n (%)**	
**Peripheral blood (PB)**	3 (2.6)
**Bone marrow (BM)**	40 (34.8)
**PB+BM**	72 (62.6)
**ECOG =1, n (%)**	104 (90.4)
** =2, n (%)**	11 (9.6)
**Regime conditioning, n (%)**	
**With DAC**	54 (47)
**No DAC**	61 (53)
**aGVHD, n (%)**	42 (36.5)
**cGVHD, n (%)**	20 (17.4)
**Disease progression, n (%)**	23 (20)
**Transplantation related mortality, n (%)**	11 (9.6)

IPSS, International Prognostic Scoring System; IPSS-R, Revised International Prognostic Scoring System; HMA, Hypomethylating agents; CR, complete remission by IWG 2006 criteria; HSCT, hematopoietic stem cell transplantation-; HLA, human leukocyte antigen; ECOG, eastern cooperative oncology group; DAC, decitabine; a/c GVHD, acute/chronic graft versus host disease; MRD, molecular residual disease.

This study was performed in accordance with the principles of the Declaration of Helsinki. Written informed consent for the submission of data to our database was routinely obtained when a patient was admitted to our center.

### Gene Sequencing and Flow Cytometry MRD Detection

The bone marrow specimens from 181 patients were detected by NGS at initial diagnosis and 115 patients (63.5%) had at least one mutation. NGS was performed in MDS patients using an Illumina MiSeq system (San Diego, CA). At initial diagnosis, an Ion AmpliSeq library including 51 common hematological disease-associated genes was constructed and tested using the ABI Ion Torrent S5 sequencer. The Ion S5 system was used to evaluate the panel of 51 common variant gene targets in hematologic malignancies, including ASXL1, ASXL2, BCOR, BCORL1, BIRC3, BRAF, CALR, CBL, CEBPA, C-KIT, CSF3R, CSMD1, DNMT3A, ETNK1, ETV6, EZH2, FBXW7, FLT3, GATA2, IDH1, IDH2, IL7R, JAK1, JAK2, JAK3, KRAS, MPL, MYD88, NOTCH1, NPM1, NRAS, PAX5, PDGFRA, PDGFRB, PHF6, PI6, PIGA, PTEN, PTPN11, RUNX1, SETBP1, SETD2, SF3B1, SH2B3, SRSF2, STAG2, TET2, TP53, U2AF1, WT1, and ZRSR2. At 30 days after HSCT, we designed an NGS panel of 12 common variant gene targets (ASXL1, ETV6, EZH2, IDH1, IDH2, NRAS, CBL, RUNX1, SF3B1, SRSF2, TET2, and TP53), and all the 115 samples were verified by panel NGS. The list of targeted genes was compiled from the reported studies (16–18) and mutation frequency in our cohort. NGS amplicons have an average gene coverage of 98.03% and an average sequencing depth of 2500. Pathogenic mutation sites are mainly based on the COSMIC database and reported literature.

As a routine clinical test, bone marrow aspirates were obtained in all patients at diagnosis and follow-up. We set a 10-color panel of nine markers including CD45, CD34, CD117, CD13, CD33, CD19, CD10, HLA-DR, CD38, and one marker of CD2, CD3, CD7, CD56, CD15, CD64, CD11b, and CD14 to analyze MRD. We analyzed on the CD45/SSc scatter plot. Flow cytometry analysis was carried out *via* Beckman Coulter (Navios, BECKman-coulter). All antibodies were obtained from Beckman Coulter Company (Navios, BECKman-coulter). Cells with abnormal expression patterns, change of expression intensity (e.g., low expression and over expression), and aberrant expression were regarded as MRD. When abnormal cells were identified, the cells were quantified as a percentage of total CD45+ cell events.

### Transplantation Regimen

Bu/Cy treatment consisted of IV Ara-c 2 g/m^2^/d on days -9 to -8, IV Bu 3.2 mg/kg/d from day -7 to day -5, IV cyclophosphamide (CTX) 1.8 g/m^2^/d from day -4 to -3, and oral semustine 250 mg/m^2^/d on day -10. Flu/Bu treatment consisted of IV Bu 3.2 mg/kg/d on days -7 to -5 and IV Flu 30 mg/m^2^/day from day -6 to day -2. TBI/Cy treatment consisted of TBI (12 Gy on days -8 to -6), IV CTX 1.8 g/m^2^/d from day -4 to approximately day -3, and oral semustine 250 mg/m^2^/d on day -9. CBA treatment consisted of IV Ara-c 2 g/m^2^/d on days -6 to -2, IV Bu 3.2 mg/kg/d from day -6 to day -3, and IV cladribine 10 mg on days -6 to -2. Decitabine application prior to HSCT, which was applied by 20 mg/m^2^/day for 3-5 days before MAC conditioning.

Anti-thymocyte globulin (ATG, 2.5 mg kg−1 day−1, days −5 to −2) was administered in the human leukocyte antigen (HLA)-haploidentical related donor (haplo-RD) and unrelated donor groups. In addition, patients received cyclosporine A, mycophenolate mofetil, and short-term methotrexate for graft-versus-host disease (GVHD) prophylaxis.

### Supportive Care and Post-Transplantation Management

Infection prevention included selective gut decontamination (oral levofloxacin, albendazole, and fluconazole) before conditioning and prophylactic anti-infection agents during the immunosuppressive period. Sinusoidal obstruction syndrome was prevented by heparin and prostaglandin E1. Other supportive therapies post-HSCT included G-CSF and IL-11 for accelerating the recovery of neutrophils and platelets, IVIG for decreasing the risk of viral infections, and irradiated blood products for maintaining a hemoglobin level above 60 g/L and a platelet count over 20 × 10^9^/L.

According to the transplantation protocol at our center, the chimerism of donor cells in peripheral blood was assessed weekly after the engraftment of neutrophils by multiple fluorescent STR analysis during the hospital stay, and the presence of CMV and EBV viremias was detected by real-time PCR. BM puncture was performed monthly to evaluate remission status in the first 3 months and then every 3 months until at least 1 year post-HSCT. Additional assessments were performed when clinically indicated. BM samples were used for FCM MRD and mutation detection. For post-transplantation bone marrow samples, we performed Sanger sequencing for a subject’s unique set of trackable mutations. The genetic mutation and FCM MRD monitoring were performed 30 days after transplantation.

### Definitions

Mutation positive (MUT^pos^) and mutation-negative (MUT^neg^) were defined as the persistence and disappearance of initial mutations when assessed at day +30 after transplant, respectively. The FCM MRD results at day +30 after transplant was divided by the value of 0.1%. The FCM‐MRD was considered high level if ≥ 0.1% (FCM^high^), low level if < 0.1% (FCM^low^).

All patients were reclassified by their IPSS-R score pre-HSCT. Sustained engraftment was defined as sustained neutrophil recovery (neutrophil recovery was the first of 3 consecutive days with a count ≥0.5×10^9^/L). Disease progression was predefined by the presence of bone marrow blasts at least 5% in morphological analysis or evidence of extramedullary sites and the necessity of any interventions due to decreased chimerism. Overall survival (OS) was defined as the time from transplantation until death from any cause or until censoring at the time that the patient was last known to be alive. Progression-free survival (PFS) was defined as the probability of being alive and free of disease progression at a given point in time. Non-relapse mortality (NRM) was defined as death after HSCT without disease progression or relapse.

### Statistical Analysis

Descriptive statistics were used to summarize and compare the demographic, disease, and clinical characteristics of the subjects. Fisher’s exact or the chi-squared test was used to compare categorical variables. A two-sample t-test or Mann-Whitney U test was used to compare continuous variables. To assess post-HSCT outcomes, we conducted a competing risk analysis, which calculates the cumulative incidence of disease progression in the presence of competing risks (nonrelapse death). The clinical or disease characteristics found to have a significant association with disease progression in univariable analysis (P < 0.10) were included in the multivariate model. PFS and OS were compared among four subjects using the Kaplan-Meier method and the log-rank test. For the progression-free survival analyses, subjects alive without any evidence of disease progression were censored at the time of last follow-up (median 15.5 months after HSCT; minimum 1 month), and end-point events were identified as disease progression or NRM. Statistical significance was defined as a P-value < 0.05. All statistical analyses were performed using SPSS version 26.0 and R 3.6.0.

## Results

### Patients and Disease Characteristics

In the presented study, the cytogenetics of all patients at diagnosis were classified as very good/good in 69 (60%) patients, intermediate in 35 (30.4%) patients, and poor/very poor in 11 (9.6%) patients. Patients were grouped before initial treatment by IPSS-R score into low-risk patients (7; 6.1%), intermediate-risk patients (32; 27.8%), high-risk patients (47; 40.9%), and very high-risk patients (29, 25.2%). The Eastern Cooperative Oncology Group (ECOG) performance status subdivided patients in two groups (ECOG=1, n =104, 90.4%; ECOG=2, n=11, 9.6%). The median (range) time from diagnosis to HSCT was 0.4 months (0.6–25.8 months). In total, 43 (37.4%) patients received induction chemotherapy, 50 (43.5%) patients received hypomethylating agents (HMAs) before HSCT, and 59 (51.3%) patients achieved CR prior to HSCT.

### DNA Sequencing and Mutation Analysis

**Figure 1:**
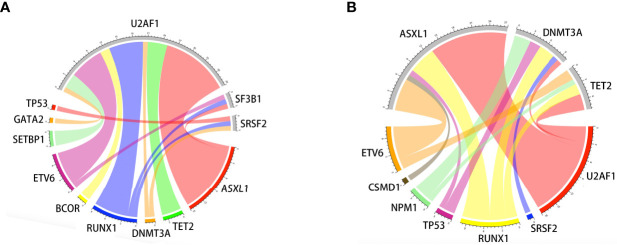
Associations of gene mutations in the MDS patient cohort outlined by a Circos diagram. Mutations in genes of the splicing machinery (U2AF1, SF3B1, and SRSF2) were associated with ASXL1 (gene belonging to the epigenetic regulation). Splicing machinery genes were mutually exclusive **(A)**. Mutations in genes of epigenetic regulation were associated with RUNX1 (gene belonging to transcriptional factors) **(B)**.

Oncogenic mutations were identified in 39 genes in 115 patients. The median (range) of VAF was 37.95% (4.6%-49.7%) at diagnosis. U2AF1 was the most frequently mutated gene (30.4%), followed by ASXL1 (24.3%), RUNX1 (16.5%), DNMT3A (10.4%), NRAS (8.7%), ETV6 (7.8%), TET2 (7.8%), NPM1 (7.8%), and TP53 (6.1%). The median number of mutations per patient was 2 (range, 1-6). Furthermore, U2AF1 gene mutations belonging to the splicing machinery were associated with ASXL1. Among splicing machinery genes, the U2AF1, SF3B1, and SRSF2 genes were mutually exclusive (**Figure 1**).

We examined the hazard ratio (HR) of death associated with the nine mutated genes. In this univariable analysis, only mutations in TP53 were associated with an increased probability of shorter OS (HR, 3.836; 95% CI, 1.109~13.269; P=0.034). TP53(HR, 3.946; 95% CI, 1.369~13.374; P=0.011) and DNMT3A (HR, 2.291; 95% CI, 0.993-5.289; P=0.052, **Table 2**) mutations were also associated with shorter PFS.

**Table 2:** Genetic mutations at diagnosis for survival outcomes.

Genetic mutation	UnivariateP value; HR (95%CI)		Multivariable P value; HR (95%CI)
	Overall Survival	Progression-Free Survival	Progression-Free Survival
ASXL1	0.768; 0.847 (0.281-2.554)	0.783; 1.113 (0.519-2.388)	–
DNMT3A	0.180; 2.131 (0.705-6.438)	0.052; 2.291 (0.993-5.289)	0.041; 2.399 (1.035-5.563)
NPM1	0.165; 2.398 (0.673-8.244)	0.291; 1.758 (0.617-5.004)	–
TET2	0.387; 0.043 (0.00-53.161)	0.241; 0.304 (0.042-2.226)	–
U2AF1	0.341; 0.585 (0.194-1.764)	0.252; 0.629 (0.284-1.390)	–
NRAS	0.366; 0.043 (0.00-39.903)	0.235; 0.043 (0.00-7.824)	–
RUNX1	0.202; 0.269 (0.036-2.017)	0.706; 0.833 (0.233-2.155)	–
ETV6	0.402; 0.044 (0.00-66.889)	0.784; 1.181 (0.360-3.878)	–
TP53	0.034; 3.836 (1.109-13.269)	0.011; 3.946 (1.369-11.374)	0.008; 4.180 (1.441-12.122)

### Outcomes and Prognostic Value of FCM and Mutation Analysis After HSCT

All 115 patients underwent HSCT; during a median post-transplantation follow-up time of 15.9 months (2.2–39.9 months), disease progression occurred in 22 (19.1%) patients, and the median (range) time from HSCT to relapse was 5.1 months (2.2–27 months). In total, 11 (9.6%) patients died from non-disease-associated and transplant-related causes (4 from infection, 4 from graft-vs-host disease, and 3 from other causes). Of the 22 patients with disease progression, no patients received secondary transplantation until the end of follow-up. There were nine patients who transformed to acute leukemia after transplantation. They received the treatment of HMA combined chemotherapy, donor lymphocyte infusion (DLI), and bcl-2 inhibitors (Venetoclax), and the status of five cases was well in hand by salvage treatments. Another 14 patients presented decreased chimerism, and these patients received maintenance therapy of HMA.

All patients achieved sustained engraftment, with median times to neutrophil and platelet engraftment of 12 (range 9-28) days and 13 (range 9-30) days, respectively. The probability of OS, PFS, and NRM after HSCT was 85.2%, 74.8%, and 9.6% at 1 year and 84.3%, 72.2%, and 9.6% at 2 years, respectively. The cumulative incidence (CI) of grade III-IV aGVHD within 100 days was 15.6%. The CI of cGVHD was 17.3% at more than 100 days after transplantation.

**Figure 2:**
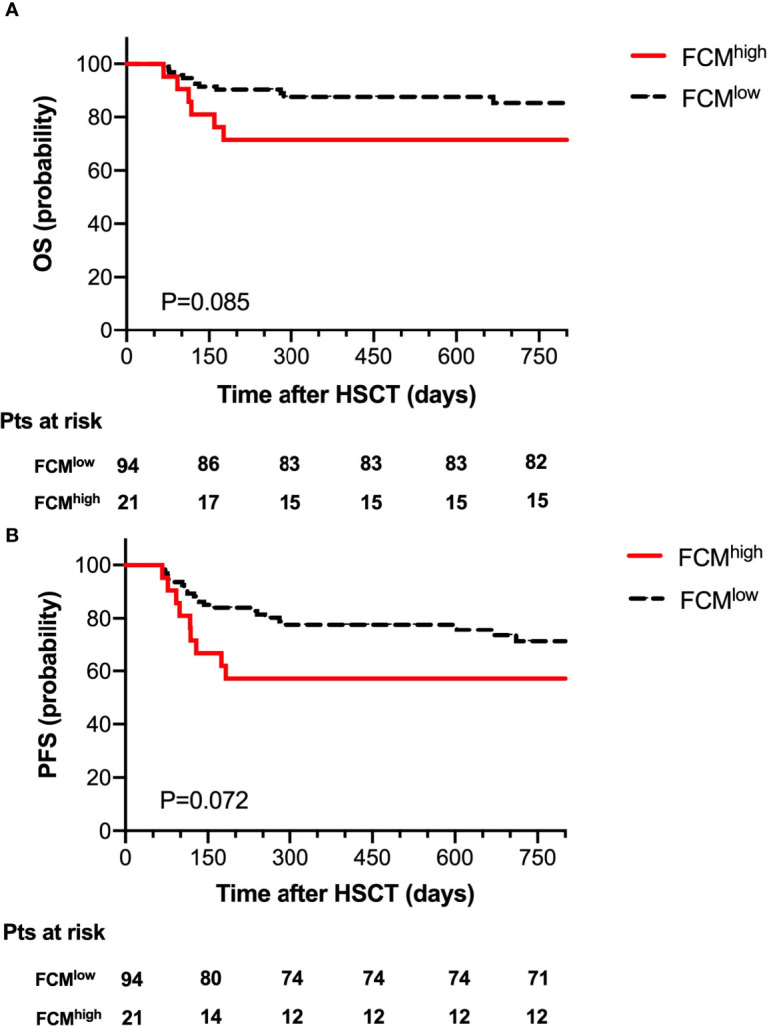
Overall survival **(A)** and progression-free survival **(B)** for 115 patients according to FCM MRD.

According to the level of MRD as determined by FCM at 30 days post-transplantation, we divided the 115 patients into an FCM^high^ group (n=21) and an FCM^low^ group (n=94). The probability of OS at 2 years after HSCT was 71.4% in the FCM^high^ group and 87.2% in the FCM^low^ group; 2-year NRM was 4.8% in the FCM^high^ group, and 10.6% in the FCM^low^ group. There was no significant difference between the probability of OS and NRM. Of note, we observed a trend toward the decreased probability of 2-year PFS in the FCM^high^ group (57.1% vs. 75.5, P=0.072, **Figure 2**).

**Figure 3:**
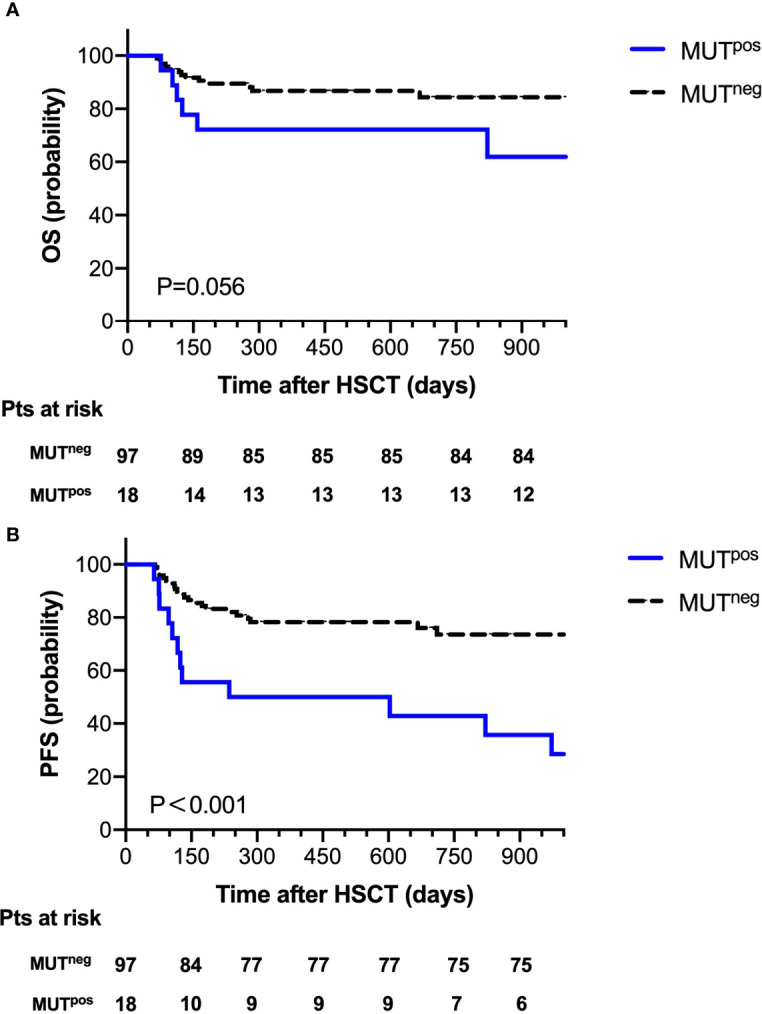
Overall survival **(A)** and progression-free survival **(B)** for 115 patients according to mutation clearance analysis.

At 30 days post-HSCT, there were 18 patients with persistent mutations. Comparing the prognostic outcome between the MUT^pos^ group (n=18) and the MUT^neg^ group (n=97), the OS at 2 years after HSCT was 72.2% in the MUT^pos^ group and 86.2% in the MUT^neg^ group; 2-year NRM was 11.1% in the MUT^pos^ group and 9.3% in the MUT^neg^ group. Notably, there was a significant difference in the probability of 2-year PFS between the MUT^pos^ group and the MUT^neg^ group (44.4% vs. 77.3%, P=0.001, **Figure 3**).

### Combination of FCM and Mutation Analysis for the Prediction of Disease Progression

To further explore the clinical impact of mutation clearance and FCM MRD analysis, we further divided all patients into four groups as follows: 5 (4.3%) patients as FCM^high^ MUT^pos^, 13 (11.3%) patients as FCM^low^ MUT^pos^,16 (13.9%) patients as FCM^high^ MUT^neg^, and 81 (70.4%) patients as FCM^low^ MUT^neg^.

**Figure 4:**
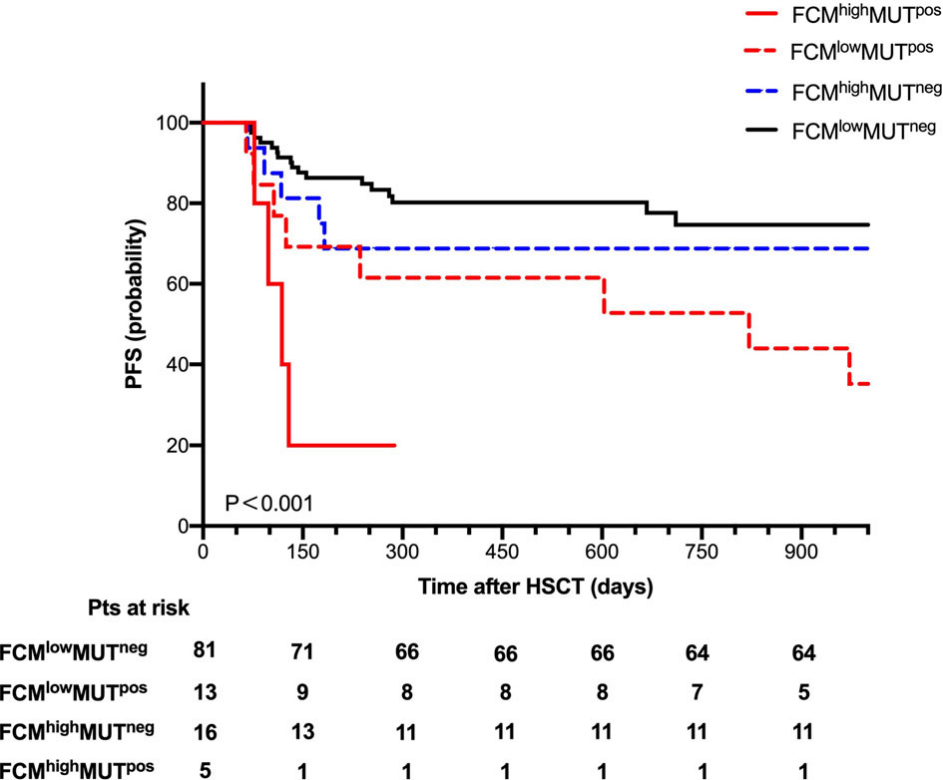
Progression-free survival for 115 patients according to combine FCM MRD with mutation clearance analysis.

The probability of OS 2 years after HSCT was 60%, 69.2%, 75%, and 88.9% (P=0.016) and of PFS at 2 years was 20%, 53.8%, 68.8%, and 79% (P < 0.001, **Figure 4**) in the FCM^high^ MUT^pos^, FCM^low^ MUT^pos^, FCM^high^ MUT^neg^, and FCM^low^ MUT^neg^ groups, respectively. Moreover, there were no significant differences in the cumulative incidence of NRM at 2 years after HSCT among these groups.

### Univariate and Multivariate Analysis of Survival Outcomes

To further verify the predictive value of the combination of FCM MRD and mutation clearance analysis for progression after HSCT, we conducted a univariate Cox regression analysis that included age (<40 years vs. ≥40 years), IPSS-R score and cytogenetic risk at diagnosis, mutations in DNMT3A and TP53, disease status before HSCT (CR vs. non-CR), donor type (HLA matched vs. unmatched), ECOG score (n=1 vs. n=2), the source of stem cells (PB vs. BM vs. PB+BM), the use of decitabine in conditioning (use vs. non-use), IPSS-R score at transplantation (<4 vs. ≥4), grade III-IV aGVHD and extensive cGVHD after transplantation, and the combined assessment of FCM MRD and mutation clearance (FCM^high^ MUT^pos^ vs. FCM^low^ MUT^neg^) after transplantation. We found that the unadjusted hazard ratio (HR) for disease progression was 7.801 (95% CI, 1.025~23.783; P < 0.001) in the FCM^high^ MUT^pos^ group (vs. FCM^low^ MUT^neg^). The other significant predictors of disease progression were IPSS-R scores ≥4 at transplantation (HR, 5.061; 95% CI, 1.546~16.572; P=0.007), DNMT3A mutations (HR, 2.291; 95% CI, 0.993~5.289; P=0.052), TP53 mutations (HR, 3.946; 95% CI, 1.369~11.374; P=0.011), and poor and very poor IPSS-R cytogenetic risk at diagnosis (HR, 4.906; 95% CI, 2.092~11.509; P<0.001). These five variables were further included in a multivariable competing risk model, which confirmed that FCM^high^ MUT^pos^ at day 30 (HR, 5.198; 95% CI, 1.408~19.195; P=0.013), IPSS-R scores ≥4 at transplantation (HR, 4.488; 95% CI, 1.336~15.080; P=0.015), poor and very poor IPSS-R cytogenetic risk at diagnosis (HR, 3.061; 95% CI, 1.041~8.730; P=0.042), and DNMT3A mutations (HR, 2.385; 95% CI, 1.005~5.664; P=0.049) were independent prognostic predictors of disease progression (**Table 3**).

**Table 3:** Univariate and multivariate analysis of prognostic factors for disease progression.

Variable	Univariate P value; HR (95%CI)	Multivariable P value; HR (95%CI)
**Progression-Free Survival**		
IPSS-R cytogenetic risk at diagnosis (poor, very poor vs. very good-intermediate)	<0.001; 4.906 (2.092-11.509)	0.042; 3.016 (1.041-8.730)
DNMT3A	0.052; 2.291 (0.993-5.289)	0.049; 2.385; (1.005-5.664)
TP53	0.011; 3.946 (1.369-11.374)	–
Score by IPSS-R at pre-HSCT (≥4 vs. <4)	0.007; 5.061 (1.546-16.572)	0.015; 4.488 (1.336-15.080)
FCM^low^MUT^neg^		
vs. FCM^high^MUT^pos^	<0.001; 7.801 (1.025-23.783)	0.013; 5.198 (1.408-19.195)
vs. FCM^high^MUT^neg^	0.334; 1.636 (0.603-4.437)	0.098; 2.375 (0.851-6.627)
vs. FCM^low^MUT^pos^	0.013; 2.940 (1.257-6.877)	0.011; 3.122 (1.296-7.521)
**Overall Survival**		
IPSS-R cytogenetic risk at diagnosis (poor, very poor vs. very good-intermediate)	0.001; 8.552 (1.141-64.111)	0.029; 4.695 (1.170-18.841)
DNMT3A	0.180; 2.131 (0.705-6.438)	–
TP53	0.034; 3.836 (1.109-13.269)	–
Score by IPSS-R at pre-HSCT (≥4 vs. <4)	0.001; 5.406 (1.913-15.283)	0.067; 6.808 (0.876-52.937)
FCM^low^MUT^neg^		
vs. FCM^high^MUT^pos^	0.067; 4.202 (0.904-19.532)	0.113; 1.399 (0.253-7.732)
vs. FCM^high^MUT^neg^	0.105; 2.429 (0.748-7.888)	0.045; 3.706 (1.031-13.321)
vs. FCM^low^MUT^pos^	0.140; 2.667 (0.815-8.728)	0.088; 2.950 (0.852-10.213)

Regarding the probability of OS, TP53 mutations (HR, 3.836; 95% CI, 1.109~13.269); P=0.034), poor IPSS-R cytogenetic risk at diagnosis (HR, 8.552; 95% CI, 1.141~64.111; P=0.001), IPSS-R score ≥4 at transplantation (HR, 5.406; 95% CI, 1.913~15.283; P=0.001), and FCM^high^ MUT^pos^ at day 30 (HR, 4.202; 95% CI, 0.904~19.532; P=0.067) were associated with poor survival outcomes in the univariate analysis, while only poor IPSS-R cytogenetic risk at diagnosis (HR, 4.695; 95% CI, 1.17~18.841; P=0.029) and IPSS-R score ≥4 at transplantation (HR, 6.808; 95% CI, 0.876~52.937; P=0.067) were associated with a higher risk of OS in the multivariate analysis.

## Discussion

Allo-HSCT is known to be the only curative treatment for MDSs, and the possibility of disease-free survival (DFS) after transplantation is about 30% to 50%. However, relapse occurs in 25%-43% of patients 5 years after transplantation, which is considered to be a major cause of treatment failure after allo-HSCT (19, 20). There were already some studies that reported that multiparameter flow cytometry-based MRD could be used as a prognostic marker for predicting relapse in MDS patients (3, 21–23). Although the role of FCM in the diagnosis and prognosis of MDS has been gradually recognized, the timing of MRD detection for MDS patients undergoing transplantation remains controversial. It was reported that positive FCM MRD pre-HSCT had a high risk of overall mortality in MDS patients (24), and other reports showed that monitoring FCM MRD at 30 days post-transplantation was feasible for the prediction of disease progression (3, 4). In this study, day 30 post-HSCT was used as the time point of detecting FCM MRD. It should be noted that there were few studies focusing on determining the value of FCM for predicting relapse after transplantation in MDS patients. How to define the cut-off value of MRD by FCM in MDS patients with HSCT is difficult. It was reported that a cut-off value of 0.1% might be applicable in AML patients (17). We routinely chose the value of 0.1% as the cut-off value in this study. We observed a trend toward the decreased probability of 2-year PFS in the high-level FCM MRD group (57.1% vs. 75.5, P=0.072). Given that different markers and strategies were carried out in FCM detection for MDS patients, the mutation clearance was also analyzed in the presented study.

It is known that mutant NPM1 transcript levels are significantly associated with prognosis and have been used in monitoring MRD in AML patients (25, 26). Furthermore, the persistence of leukemia-associated mutations, such as FLT3-ITD, NPM1, and CEBPA mutations, after the initial course of standard induction chemotherapy imparts a significantly increased risk of subsequent leukemic relapse and death (27–29). TaeHyung Kim et al. performed NGS with a targeted gene panel (including FLT3-ITD, DNMT3A, TET2, PTPN11) in 104 AML patients at day 21 post-transplantation, and they observed that patients with VAF post-HSCT (≥0.2%) had increased relapse incidence (56.2% vs 16.0%, P < 0.001). This study demonstrated that NGS-based post-transplantation monitoring in AML patients is feasible and could distinguish high-risk patients for relapse (16). Some gene mutations will disappear during the clone evolution, which may increase the difficulty of monitoring MRD by NGS. In addition, the application of dd-PCR (droplet digital-PCR) was limited for only one gene can be detected each time. The multi-gene panel by NGS we used partially weakened the impacts of clonal evolution. However, the current NGS methods necessitate a high VAF threshold to confirm mutations and the cut-off value has not been determined. It is essential to combine a multigene NGS assay with other methods such as flow cytometry, WT-1/EVI-1 quantitative, etc.

It should be emphasized that the distributions of gene mutations in MDS patients were different from those in AML patients. Somatic mutations involved in the epigenetic regulation and spliceosome pathways were more common and played an important role in the pathogenesis of MDS (8, 11, 18, 30, 31). U2AF1, ASXL1, RUNX1, and DNMT3A gene mutations were the most common mutations in our study and showed frequencies that were similar to those in other reports (31.2%-34.7%) (18, 32). We also found that mutations involving splicing pathways were associated with genes involved in epigenetic regulation (ASXL1, TET2, and DNMT3A), which might result from the fact that chromatin and histone modifications were both involved in pre-mRNA splicing (33). Additionally, as previously reported, RUNX1 and ETV6 mutations were associated with genes involving splicing pathways and epigenetic regulation (18, 34). However, only the patients with TP53 mutations had a significantly increased probability of shorter OS (P=0.034) and PFS (P=0.011), and patients with DNMT3A mutations showed a trend toward shorter PFS (P=0.052).

Whether the presence of gene mutations could be used as an MRD monitoring marker for MDS patients receiving either chemotherapy or transplantation needs to be further studied. Based on the persistence of these gene mutations at day 30 after transplantation, we observed the significant difference in the probability of PFS at 2 years between the MUT^pos^ group and the MUT^neg^ group (44.4% vs. 77.3%, P < 0.001), which indicated that mutation clearance had predictable value for disease progression. When we combined FCM MRD with gene clearance as the early MRD marker after transplantation, we found that the probability of PFS at 2 years after HSCT was 79% in the FCM^low^ MUT^neg^ group, while it was 20% in the FCM^high^ MUT^pos^ group. Further multivariate analysis verified that FCM^high^ MUT^pos^ was an independent risk factor for disease progression. To our knowledge, our study shows for the first time that the combination of FCM and mutation clearance is a useful tool for monitoring disease in MDS patients post-HCST. It was previously reported that 1-month WT-1 expression after HSCT could predict subsequent relapse in MDS patients (15), and XS Zhao et al. reported that the combined use of WT-1 and flow cytometry monitoring could promote the sensitivity of the prediction of relapse after HSCT in acute leukemia patients (4). Since WT-1 gene expression was not the specific marker for leukemia or MDS in patients, we speculated that a target gene panel for MRD would be more accurate. Our data also suggested that the combination of NGS and flow FCM MRD detection has 82.6% sensitivity for predicting disease progression, which was higher than the previous reports (4, 15).

There were a few reports on the prognostic value of recalculated IPSS-R scores before transplantation. In a pioneer study, compared to the IPSS-R score at diagnosis, the MDS patients were regrouped as improved, worsened, and unchanged according to IPSS-R score at transplantation. However, event-free survival (EFS) was not statistically significantly different among these groups (35). In the presented study, we found that an IPSS-R score ≥ 4 pre-HSCT (HR, 4.488; P=0.015) could predict lower PFS. Notably, blast counts (<5% and ≥5%) before transplantation had no significant impact on PFS, while poor and very poor cytogenetic IPSS-R was a risk factor of PFS after transplantation. Despite a relatively small sample size, our study still suggested that regrouping by IPSS-R score at HSCT could be necessary for the MDS patients before transplantation.

In summary, our data strongly indicated that the monitoring of MRD by both FCM and gene mutation clearance at day 30 could help in the prediction of relapse of MDS patients after myeloablative transplantation. The pooling of more patients in a well-designed clinical trial may further demonstrate the predictive value of the combined MRD monitoring strategy in clinical practice.

## Data Availability Statement

The original contributions presented in the study are publicly available. This data can be found here: https://doi.org/10.6084/m9.figshare.14724681.v1.

## Ethics Statement

Written informed consent was obtained from the individual(s) for the publication of any potentially identifiable images or data included in this article.

## Author Contributions

DW and YX contributed to the conception of the study and manuscript revision. CH, MY and LZ contributed to collecting and performing data analysis and preparing the manuscript. SJ, HS, and MZ helped collect and perform data analysis and prepare the manuscript. JC and MM contributed to data analysis and manuscript revision. All authors contributed to the article and approved the submitted version.

## Funding

This work was supported by grants from the National Natural Science Foundation of China (81730003, 81870120, 82070187), the Natural Science Foundation of Jiangsu Province (BK20171205), the social development project of Jiangsu Province (BE2019655), the Priority Academic Program Development of Jiangsu Higher Education Institutions (PAPD), and the National Key Research and Development Program (2016YFC0902800, 2017YFA0104500, 2019YFC0840604).

## Conflict of Interest

The authors declare that the research was conducted in the absence of any commercial or financial relationships that could be construed as a potential conflict of interest.

## Publisher’s Note

All claims expressed in this article are solely those of the authors and do not necessarily represent those of their affiliated organizations, or those of the publisher, the editors and the reviewers. Any product that may be evaluated in this article, or claim that may be made by its manufacturer, is not guaranteed or endorsed by the publisher.
